# SAXS Examinations of the Redox-Dependent Formation of a DNA-SOD1 Complex

**DOI:** 10.3390/ijms232012673

**Published:** 2022-10-21

**Authors:** Huiling Wang, Mingfang Wang, Zefeng Nie, Shuang Qiu, Xiaoping Huang, Xiang Li, Yanfang Cui, Chunrong Liu, Changlin Liu

**Affiliations:** Key Laboratory of Pesticide and Chemical Biology of Ministry of Education, School of Chemistry, Central China Normal University, Wuhan 430079, China

**Keywords:** SOD1, DNA, SAXS, redox-dependent binding, HADDOCK docking

## Abstract

Cu/Zn superoxide dismutase (SOD1) plays a key role in the maintenance of cellular reactive oxygen species (ROS) homeostasis as an antioxidant enzyme. We recently found that SOD1 is involved in the regulation of gene expression in response to changes in cellular ROS levels by binding to DNA-specific sequences. Moreover, the SOD1 binding to DNA was observed to be redox-dependent in solutions. Thus, we examined the redox-dependent DNA binding of SOD1 by multiple measurements, including small-angle X-ray scattering (SAXS), indicating the redox-dependent formation of a DNA-SOD1 complex in solutions. The redox-dependent formation of the DNA-SOD1 complex could underlie the SOD1 regulation of gene expression.

## 1. Introduction

Cu/Zn superoxide dismutase (SOD1) is a 32 kDa homodimeric enzyme, with each subunit holding Cu^2+^ and Zn^2+^ in close proximity [1] and is distributed throughout the cytosol, nucleus, and mitochondrial intermembrane space [2]. What we are familiar with is that SOD1 is a key antioxidant enzyme, with the main function of catalyzing the disproportionation of O_2_^•−^ to O_2_ and H_2_O_2_ [3], maintaining cellular reactive oxygen species (ROS) homeostasis [4]. Increasing evidence shows that SOD1 is involved in redox signaling networks to regulate cell growth and metabolic pathways [5], integrate signals from oxygen and glucose to repress respiration [6], and modulate ribosome biogenesis in KRAS-mutant non-small-cell lung cancer [7]. Moreover, we found that the specific inhibition of SOD1 in cancer and normal cells represses the signaling pathways and their crosstalk that supports cancer cell growth but stimulates the signaling network that promotes cancer cell cycle arrest and apoptosis [8,9].

Multiple tests and bioinformatic analyses performed a decade ago demonstrated that SOD1 binds DNA in solutions, and this binding is mainly driven by electrostatic interactions between SOD1 and DNA [10,11,12,13,14,15]. Moreover, the hydrogen peroxide (H_2_O_2_)-mediated high nucleus distribution of SOD1 showed that SOD1 acts as a DNA-binding protein capable of linking to the regulation of gene expression in yeast [16]. In fact, our recent results revealed a lot of DNA sequences specifically associated with SOD1, and SOD1 is known to regulate gene expression in response to changes in the levels of H_2_O_2_ in mammalian cells by binding to DNA [17]. The base GGA triads were observed to have direct contact with SOD1 in the DNA binding. Moreover, the SOD1 binding to DNA was found to be redox-dependent in solutions. These findings both in solutions and cells prompted us to further examine whether the formation of a DNA-SOD1 complex is redox-dependent in solutions or not.

Here, we further examined the binding to a synthetic double-stranded DNA fragment 5’-ATGGAATGGAAT-3’ (dsDNA) of SOD1 and the formation of their complex in solutions by multiple measurements including small-angle X-ray scattering (SAXS). The profiles of the DNA-SOD1 complex formed in a redox-dependent manner were produced and compared in solutions by fitting the SAXS data into the optimized model obtained by HADDOCK.

## 2. Results and Discussion

### 2.1. Determination of the Binding Constant of SOD1 to dsDNA

**Figure 1:**
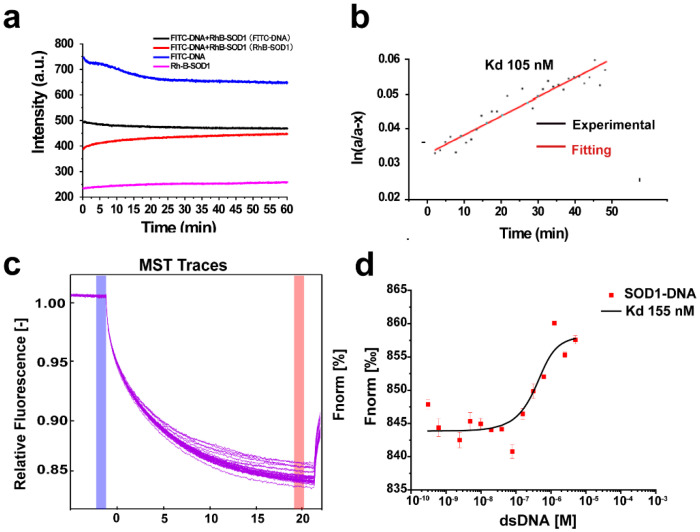
FRET and MST determinations of the dsDNA binding of SOD1. (**a**) 100 μM FITC-dsDNA and 200 μM RhB-SOD1 were mixed and immediately excited at 450 nm in 20 mM Tris-HCl (pH 7.4). (**b**) The binding affinity (*K*_d_) of SOD1-RhB for FITC-dsDNA was calculated based on kinetic curves. (**c**) MST traces of SOD1 and DNA. (**d**) Binding of SOD1-RhB to dsDNA and fitting of affinity (*K*_d_). Samples were tested in the red channel by MST; excitation power: auto-detect 20%, MST power: medium. MST data were processed using MO. Control V1.6.1 and MO. Affinity Analysis V2.3.

The bovine and human (Figure S1) SOD1 binding to dsDNA has been examined by an electrophoretic mobility shift assay (EMSA) and fluorescence anisotropy [17]. Here, the binding constant of SOD1 to dsDNA was further determined by both fluorescence resonance energy transfer (FRET) and microscale thermophoresis (MST) assays. First, FRET was used to determine the binding constant of bovine SOD1 to dsDNA. The fluorophores rhodamine B (RhB) [18] and fluorescein-5-isothiocyanate (FITC) [19] were linked to SOD1 and DNA, respectively, in the FRET examination because FRET can occur between this pair of fluorophores in a distance of ≤ 10 nm [20]. The FRET effect was observed between the modified DNA and SOD1 (Figure S2a), indicating that the SOD1 binding of dsDNA occurred in the solution. Moreover, the time profiles showed that the change in the FRET fluorescence intensity became constant after 60 min (Figure 1a). The binding constant (*K*_d_) of SOD1 to dsDNA was fitted to be 105 nM based on the kinetic curve (Figure 1a,b). Then, the SOD1 binding constant of dsDNA was also determined by MST [21]. The recombinant human SOD1 was labeled with the dye RED-NHS according to the commercial kit instructions for the MST (Monolith NT.115) measurements (Figure S2b). The binding check tests indicated that the signal/noise (10.4) was large enough to conclude the SOD1 binding of dsDNA (Figure S2c). The MST data confirmed the dsDNA binding of SOD1 with a binding constant of *K*_d_ = 155 nM (Figure 1c,d). These results indicated the formation of a stable DNA-SOD1 complex through DNA–protein interactions in the solution. In fact, the early fluorescence polarization assays showed a strong association of SOD1 with the FAM-labeled dsDNA in solutions [17]. The difference among the three *K*_d_ values can be attributed to the uses of different assay methods and conditions, as well as to the influence of the respective labeling of the enzyme and dsDNA on their association.

### 2.2. SAXS Characterization of the Mixture of SOD1 and dsDNA

To further confirm the association between SOD1 and dsDNA, the mixtures of the SOD1 and dsDNA complex were examined and compared with SAXS in solutions under varied redox conditions. SAXS allows for a full sampling of the conformational space of a large biomolecular complex in solutions without requiring any chemical modifications to any components in the complex, although SAXS characterization can only provide contour information for the complex with a low resolution of 10–50 Å [22,23]. Moreover, there were no constraints imposed on the compositions of the solutions. SAXS data can reflect the profiles of the solution complexes with size scales of 50–500 Å [24,25], a good match with the major features present in DNA–protein complexes. Therefore, SAXS has the potential to examine solution structures of DNA–protein complexes and to reflect variances in the solution profiles of DNA–protein complexes formed under varied conditions. However, the interpretation of typical SAXS curves for DNA–protein complexes is challenging because scattering signals from DNA and proteins, as well as their nonlinear combinations stemming from the interactions between the DNA and proteins (also called cross-term), all contribute to what is measured [26,27]. Fortunately, we can examine and compare the profiles of the dsDNA-SOD1 complex and the significant changes in the electron density of the complex formed under varied redox conditions using SAXS curves, thereby confirming the redox-dependent formation of the dsDNA-SOD1 complex. Moreover, comparative SAXS determinations can provide changes in the redox-dependent profiles of the dsDNA-SOD1 complex in solutions when detailed models are available for both the comparison and fitting of the data.

**Figure 2:**
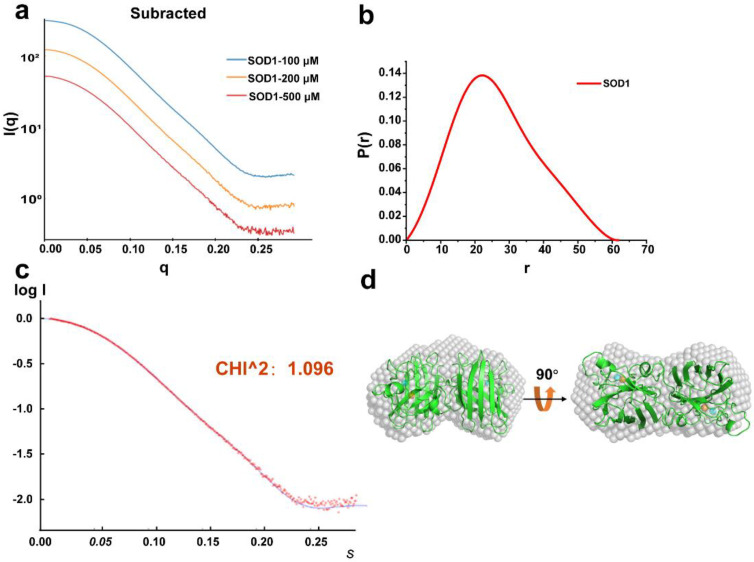
SAXS analysis of SOD1. (**a**) The concentration−dependent scattering profiles of SOD1. These scattering profiles were collected, respectively, at 100, 200, 500 μM SOD1. (**b**) Distance distribution function *P(r)* of SOD1. (**c**) Fitting of the SAXS data with the X−ray SOD1 structural data. (**d**) The structural model of SOD1 (white) calculated using the SAXS data was mapped onto the crystal structure of SOD1 (green, 1CBJ).

To determine the reliability of the SAXS characterization of the dsDNA-SOD1 complex in solutions, SAXS data of bovine SOD1 (Figure 2a and Figure S3) were first acquired at three concentrations (100, 200, and 500 μM) without any oxidants or reducing agents (pH 7.4, 37 °C) and analyzed by programs including BioXTAS RAW [28]. We converted the SAXS data *I(q)* from reciprocal to real space (Figure 2b) by primusqt [29] using GNOM [30] to compute the pair distance distribution function *P(r)*. The quality of this computation can be assessed by three calculations. First, two SAXS parameters, i.e., the radius of the gyration (Rg) and maximal dimension (D_max_), which characterize the profile of SOD1, were 20.6 ± 0.3 Å and 61 Å (Table 1), respectively, consistent with the previously reported values [31]. Then, the theoretical SAXS curve (lg*I(q)*) obtained using both the X-ray SOD1 structural data in PBD and the software CRYSOL [32] overlapped well with the experimental SAXS curve with χ^2^ = 1.096 (Figure 2c). Moreover, the spherical model of SOD1 provided by the SAXS curve matched well with the SOD1 crystal structures (Figure 2d). Finally, the back computation from *P(r)* to *I(q)* indicated that the computed *I(q)* curve coincided with the measured one (Figure 2a). Therefore, the SAXS characterization reliably reflected the redox-dependent formation and profile changes in the dsDNA-SOD1 complex in solutions.

**Figure 3:**
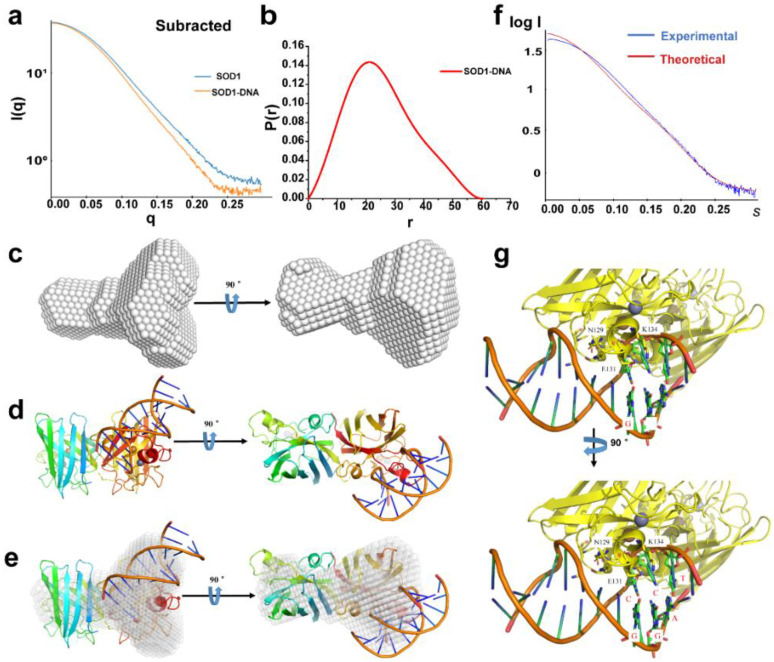
The SAXS analysis of the dsDNA-SOD1 complex. (**a**) Scattering profiles of SOD1 and dsDNA-SOD1 complex under tested conditions. (**b**) *P(r)* distribution functions of SOD1 and dsDNA-SOD1 complex. (**c**) The profile of the dsDNA-SOD1 complex provided using the SAXS data. (**d**) The optimized structural model of the dsDNA-SOD1 complex provided by HADDOCK based on the conformation stemming from the SAXS data. (**e**) The SAXS profile of the dsDNA-SOD1 complex merged with the HADDOCK model. (**f**) Fitting of the dsDNA-SOD1 complex SAXS data with the HADDOCK model of the dsDNA-SOD1 complex. (**g**) The proposed interaction sites between SOD1 and dsDNA.

According to the SAXS characterization of SOD1, the SAXS data were recorded and processed for the mixture containing bovine SOD1 and dsDNA of 100 μM after 24 h incubation under the same conditions (Figure 3a,b and Figure S4). The SAXS curve of this mixture did not overlap that of SOD1, indicating that the SOD1 structure slightly changed in the mixture. Indeed, the smaller values of R_g_ (19.36 ± 0.14 Å) and D_max_ (60.41 Å) than those of SOD1 alone (Table 1) were calculated using the SAXS curve. The profile provided by processing the SAXS data with the software package ATSAS 2.8.4 was found to be composed of two components and distinct from that of SOD1 alone (Figure 3c). Obviously, these two components are SOD1 and dsDNA, respectively, i.e., SOD1 formed a DNA–protein complex through the association in the solution, supporting the conclusion obtained by the FRET and MST assays. Moreover, the SOD1 in the complex became more compact compared to SOD1 alone, as demonstrated by the two SAXS parameters.

To understand the possible binding modes of SOD1 to dsDNA in the dsDNA-SOD1 complex, the interactions between SOD1 and dsDNA were simulated by HADDOCK [33], one of the most commonly used molecular docking platforms for DNA–protein complexes. The docking calculation showed that a dimeric SOD1 molecule can bind dsDNA in two modes: parallel to and perpendicular to the DNA double helix (Figure S5 and Figure 3d). The scores for these two binding modes indicated that the binding perpendicular to the DNA double helix was a reasonable binding mode of SOD1 to DNA [17], supported by larger interaction energy (electrostatic plus van der Waals) and larger contact areas between SOD1 and dsDNA (Figure S5). This binding mode was compatible with the profile of the dsDNA-SOD1 complex produced by the SAXS data (Figure 3c), as indicated by merging this structural model (Figure 3d) exactly with the SAXS profile (Figure 3e). Conversely, the theoretical SAXS curve obtained using the structural model was also in agreement with the experimental SAXS curve of the dsDNA-SOD1 complex (Figure 3f). The association of a dimeric protein with dsDNA in a perpendicular manner only through its one subunit was one of the interaction modes found between DNA and proteins [34].

The potential SOD1 amino acid residues that had contact with dsDNA can be proposed in the DNA-SOD1 complex according to the docking model of the complex. An inspection of the merged model (Figure 3e) found that the α helix and related loops in SOD1 penetrated the major groove and bound the base GGA triad in dsDNA [17] (Figure 3g). The SOD1 residues that had contact with GGA could be K120, N129, E131, K134, S140, and R141 because the positively charged residues K120 and R141 interacted with the negatively charged DNA phosphate backbone. The electrostatic interactions could play a key role in the formation of the DNA-SOD1 complex, as indicated by the overwhelming electrostatic energy over the van der Waals energy (Figure S5).

### 2.3. Effect of Redox Conditions on the Formation of the dsDNA-SOD1 Complex

**Figure 4:**
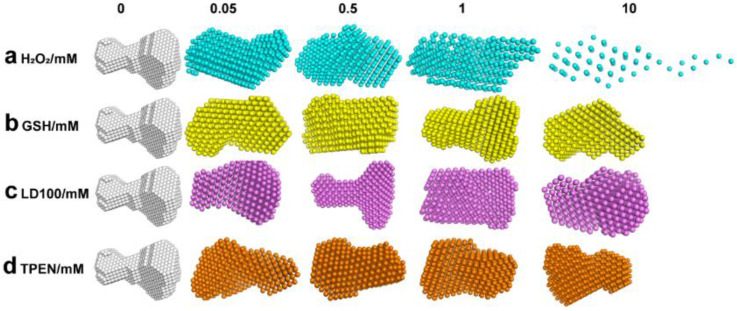
SAXS profiles of the dsDNA-SOD1 complex under varied redox conditions. Concentrations of 100 μM SOD1 and 100 μM dsDNA were incubated at 37 °C for 24 h in a 10 mM Ph 7.4 Tris-HCl buffer with H_2_O_2_, GSH, LD100, or TPEN (0, 0.05, 0.5, 1, 10 mM). Samples were centrifuged at 10,000 rpm at 4 °C for 10 minutes to remove possible aggregates before data collection.

To observe the effect of the redox conditions on the formation of the dsDNA-SOD1 complex, the SAXS data were first acquired and processed for the mixtures composed of 100 μM bovine SOD1 and dsDNA after 24 h incubation with H_2_O_2_ of varied concentrations (Figures S6a, S7a and S8a). The SAXS parameters (R_g_ and D_max_, Table 1) were found to increase as the H_2_O_2_ concentration rose (0, 50, 500, 1000 μM) compared to those of the complexes without H_2_O_2_. ATSAS processing of the SAXS data produced profiles (Figure 4a) that were completely distinct from those of SOD1 alone and the dsDNA-SOD1 complex without any redox agents (Figure 2d and Figure 3c). These results indicated that the presence of H_2_O_2_ could disrupt the SOD1 binding to dsDNA or break the complex formed in solutions. As previously reported, H_2_O_2_ can lead to the destruction of the SOD1 structure through oxidation of the amino acids involved in the SOD1 copper ion [9]. The structural destruction prevents SOD1 from binding to dsDNA and leads to the dissociation of the dsDNA-SOD1 complex. However, the presence of H_2_O_2_ did not alter the structure of redox metal-free proteins such as BSA, as indicated by the circular dichroism and SDS-PAGE tests (Figure S9).

The SAXS data were also acquired and processed for the mixture containing 100 μM SOD1 and DNA in the presence of glutathione (GSH) under the same conditions (Figures S6b, S7b and S8b). The SAXS parameters were not changed with the increasing concentrations of GSH (Table 1). The profiles of the dsDNA-SOD1 complex (Figure 4b) produced by the SAXS data were in agreement with that of the complex in the absence of oxidants and reducing agents (Figure 3c), indicating that the addition of GSH did not destroy the SOD1 structure. Therefore, the dsDNA binding of SOD1 occurred and the stable structure of the dsDNA-SOD1 complex was maintained.

To further verify the effect of the oxidation-mediated structural change in SOD1 on the formation of the dsDNA-SOD1 complex, we examined whether or not the specific SOD1 inhibitor or the most used Zn^2+^ chelator affected the dsDNA binding of SOD1. LD100 not only specifically inhibited SOD1 activity but also altered its structure by chelating the Cu^2+^ ion in SOD1 [9], and TPEN also altered the conformation of SOD1 by chelating its Zn^2+^ [35]. Thus, the addition of either LD100 or TPEN can alter not only the enzyme activity of SOD1 but also the conformation of SOD1. The SAXS data indicated that the specific inhibition led to an increased D_max_ value of the dsDNA-SOD1 complex as the concentration of LD100 increased, although their radius of gyration remained unchanged in the presence of LD100 (Table 1). Furthermore, the SAXS data showed that the profiles of the dsDNA-SOD1 complex (Figures S6c,d, S7c,d and S8c,d) were significantly altered and became notably relaxed with the increased addition of either LD100 or TPEN (Figure 4c,d) compared to that of the complex without the addition of any agents (Figure 3c). These notable alterations in the profile of the dsDNA-SOD1 complex were similar to those caused by the addition of H_2_O_2_ and can be attributed to the variations in the structure of SOD1.

## 3. Materials and Methods

### 3.1. Human SOD1 Expression and Purification

N-terminally His-tagged human SOD1 was expressed in *E. coli* BL21 (DE3) cells at 37 °C. The expression of SOD1 was induced by the addition of 1 mM IPTG, and cells were cultured at 20 °C for 20 h. An amount of 0.6 mM CuSO_4_ and ZnSO_4_ were co-incubated with the cells for the activation of SOD1. Upon centrifugation, cell pellets derived from 1 L bacterial culture were suspended, sonicated, and centrifuged in 30 mL lysis buffer (20 mM Tris-HCl, 150 mM NaCl, 10 mM PMSF, pH 8.0–8.5). The supernatant was applied to a Nickel-NTA chromatography column, washed with 2–3 column volumes of buffer A (50 mM Tris-HCl, 10 mM imidazole, pH 8.0–9.0) and buffer B (50 mM Tris-HCl, 30 mM imidazole, pH 8.0–9.0), and bound SOD1 was eluted with the elution buffer (50 mM Tris-HCl 250 mM imidazole, pH 8.0–9.0) in 2–3 column volumes. Further purification was performed using SP-Sepharose (GE Healthcare) columns. Protein purity was tested using SDS-PAGE and Western blot assay.

### 3.2. Fluorescence Resonance Energy Transfer (FRET)

The dsDNA fragment (Wuhan GeneCreate Biological Engineering Co., Ltd.) was labeled with FITC and bovine SOD1 (Sigma Aldrich) was labeled with RhB [18]. Amounts of 100 μM FITC-dsDNA and 200 μM RhB-SOD1 were mixed in 20 mM Tris-HCl (pH 7.4) and the mixtures were incubated at 37 °C for 2 h. Samples were excited at 450 nm and their emission spectra were collected in the wavelength range of 460–650 nm. Fluorescence kinetics curves were acquired at 450 nm of excitation wavelength and in the emission wavelength ranges of 510–520 nm and 570–580 nm.

### 3.3. Microscale Thermophoresis (MST) Assay

The recombinant human SOD1 was labeled with the commercial dye NHS-RED (MonolithTM RED-NHS) according to its instructions. An amount of 10 μL of 300 μM NHS-RED was added to 90 μL SOD1 (5 mg/mL, 156 mM) and the mixture was incubated for 30 min at room temperature in the dark. The commercial affinity column was washed with 3 × 10 mL H_2_O and 3 × 10 mL 10 mM PBS (pH 7.4). NHS-SOD1 was purified from the pre-balanced column and was tested by MST. The MST data were analyzed by the software MO. Control V1.6.1.

NHS-SOD1 was diluted with 10 mM PBS (pH 7.4) containing 0.05% Tween to give a final concentration of 500 nM and was mixed for 5 min with 20 μM dsDNA at room temperature. These samples were loaded into the Nano Temper Technologies glass capillaries and tested by MST. System settings: Monolith NT, 115 Capillary, excitation power of auto-detect 20%, medium MST power. The MST data were analyzed by MO. Control V1.6.1.

An amount of 20 μM dsDNA was added to each of the 16 PCR tubes with two-fold dilution and mixed with 500 nM SOD1. These 16 samples were first incubated for 5 min at room temperature and then transferred to the Nano Temper Technologies glass capillaries for the MST tests. System settings: Monolith NT, 115 capillary, excitation power of auto-detect 20%, medium MST power. The MST data were analyzed via MO. Control V1.6.1 and MO. Affinity analysis with V2.3 gave the binding affinity of NHS-SOD1 for dsDNA.

### 3.4. Small-Angle X-ray Scattering (SAXS)

To obtain the profiles of the dsDNA-SOD1 complex, SAXS experiments were carried out at the BL19U2 beamline of the National Facility for Protein Science in Shanghai (NFPS) at Shanghai Synchrotron Radiation Facility. Amounts of 100 μM bovine SOD1 and 100 μM dsDNA were mixed and incubated for 24 h at 37 °C in 20 mM pH 7.4 Tris-HCl, then centrifuged for 10 min at 10,000 rpm at 4 °C to remove potential aggregates before data collection. SAXS data were collected for 100 μL samples, following calibration of the beamline′s parameters. The X-ray wavelength was 1.03 Å, the sample–detector distance was 2.64 m, and the detector pixel size was 172 μm. Each sample was exposed 10 times to X-ray during peristalsis and the exposure time was 1 s. The program RAW was used to average the data and subtract the scattering of the buffer. Datasets were merged with PRIMUS. The real-space *P(r)* distribution, radius of gyration (Rg), and maximum distance D_max_ were calculated with GNOM. Twenty independent ab initio modeling jobs were performed using DAMMIN and averaged by DAMAVER. The theoretical curve of the crystal structure and the experimental curve were fitted with CRYSOL, and the superposition between the spherical model and crystal structure using Pymol 2.4.

### 3.5. HADDOCK Docking

The easy interface of HADDCOK 2.2 was used to generate the structural model of the dsDNA-SOD1 complex. The crystal structure of bovine SOD1 (PDB: 1CBJ) was downloaded from PDB and the dsDNA 3D structure was acquired from the 3D-DART interface of HADDOCK 2.2. All residues on the surface of SOD1 and all bases in dsDNA were set as active sites in the experimental input parameters. When the docking was finished, the lowest HADDOCK scoring structures formed a cluster that was taken as the final ensemble of the complexes. The most stable model was analyzed in PyMOL2.4.

### 3.6. Circular Dichroism Spectra

Circular dichroism (CD) spectra were measured in the wavelength range of 200–260 nm at 25 °C using a chariscan spectrometer (Applied Photophsics, Beverly, MA, USA). An amount of 100 μM BSA was incubated with 10 mM H_2_O_2_ at 37 °C for 0, 2, 4, 8, 12, 24, and 48 h, respectively, for the CD measurements.

## 4. Conclusions

SOD1 was found to be involved in the regulation of gene expression in response to changes in the cellular ROS content as a DNA-binding protein [17]. Here, the determinations of both the binding constants and DNA-SOD1 complex profiles under varied redox conditions indicated that SOD1 had a strong affinity for DNA and formed a stable DNA–protein complex. The addition of either H_2_O_2_ or SOD1 inhibitors blocked the formation of or resulted in the destruction of the DNA-SOD1 complex because these two kinds of agents can destroy or alter the structure of SOD1. However, the formation of the DNA-SOD1 complex was not influenced by the reductive solution. Obviously, these results provide a line of support for understanding at a molecular level the mechanism by which SOD1 is involved in the regulation of gene expression in response to changes in the cellular ROS content.

## Figures and Tables

**Table 1:** The forward scattering [I (0)], radius of gyration (Rg), maximal dimension (Dmax) of samples in solutions.

Conditions	Construct	Concentrations (mM)				
		0	0.05	0.5	1	10
**H_2_O_2_**	I(0) (×10^5^)	52.74 ± 0.04	64.71 ± 0.06	60.63 ± 0.07	58.02 ± 0.09	N/A
	Rg (Å)	19.36 ± 0.14	19.99 ± 0.15	19.93 ± 0.03	20.19 ± 0.04	N/A
	Dmax (Å)	60.41	67.76	67.91	69.92	N/A
**GSH**	I(0) (×10^5^)	52.74 ± 0.04	58.61 ± 0.06	57.09 ± 0.05	61.82 ± 0.06	55.86 ± 0.06
	Rg (Å)	19.36 ± 0.14	19.41 ± 0.11	19.40 ± 0.18	19.36 ± 0.18	19.35 ± 0.05
	Dmax (Å)	60.41	61.02	60.91	61.04	61.01
**TPEN**	I(0) (×10^5^)	52.74 ± 0.04	57.38 ± 0.05	59.57 ± 0.03	58.27 ± 0.06	59.14 ± 0.05
	Rg (Å)	19.36 ± 0.14	19.39 ± 0.03	19.40 ± 0.03	19.34 ± 0.04	19.34 ± 0.04
	Dmax (Å)	60.41	60.86	60.42	60.63	62.98
**LD100**	I(0) (×10^5^)	52.74 ± 0.04	56.04 ± 0.07	56.94 ± 0.07	58.50 ± 0.13	N/A
	Rg (Å)	19.36 ± 0.14	19.32 ± 0.06	19.35 ± 0.04	19.44 ± 0.06	N/A
	Dmax (Å)	60.41	61.32	63.12	67.54	N/A

## Data Availability

The data that support the findings of this study are available from the corresponding author upon reasonable request.

## Supplementary Materials

The following supporting information can be downloaded at https://www.mdpi.com/article/10.3390/ijms232012673/s1. Ref [19] was cited in the Supplementary Materials.
