# Supplementary material for: SAXS Examinations of the Redox-Dependent Formation of a DNA-SOD1 Complex
Source: Int J Mol Sci. 2022 Oct 21;23(20):12673. doi: 10.3390/ijms232012673 (PMC9604462; doi:10.3390/ijms232012673)
Supplement: Supplementary file 1 [file ijms-23-12673-s001.zip › ijms-1904696 - supplementary.pdf]

International Journal of
Molecular Sciences

MDPI

Supplementary Materials

SAXS Examinations of the Redox-Dependent Formation of a DNA-SOD1 Complex

Huiling Wang, Mingfang Wang, Zefeng Nie, Shuang Qiu, Xiaoping Huang, Xiang Li, Yanfang Cui, Chunrong Liu and Changlin Liu *

Key Laboratory of Pesticide and Chemical Biology of Ministry of Education, School of Chemistry, Central China Normal University, Wuhan 430079, China
* Correspondence: liuchl@mail.ccnu.edu.cn

Supplementary Figures

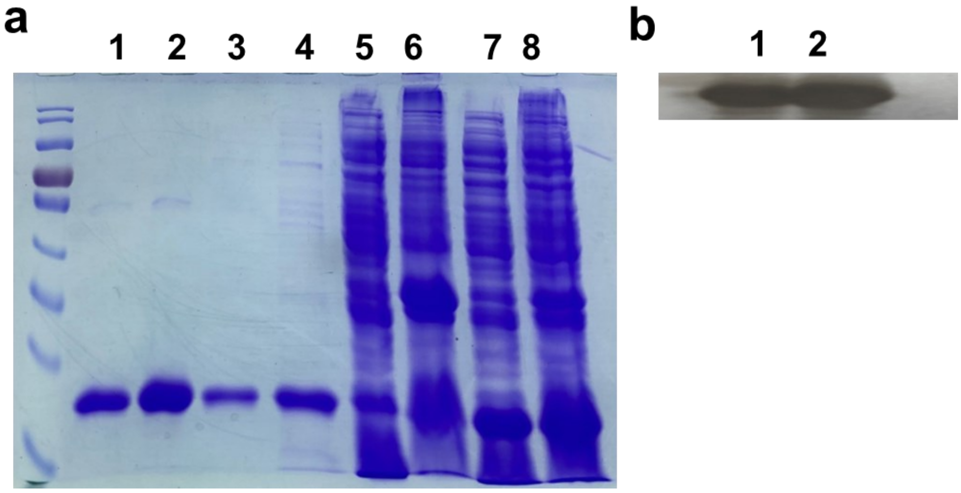

Figure S1. SDS-PAGE and western blot assay of recombinant human SOD1. (**a**) SDS-PAGE of human SOD1, line 1: elution buffer average, line 2: elution buffer max, line 3: wash buffer 2, line 4: wash buffer 1, line 5 flow solution, line 6: precipitation, line 7: supernate, line 8: bacteria solution. (**b**) western blot of human SOD1, line 1: 4 μM SOD1, line 2: 5 μM SOD1.

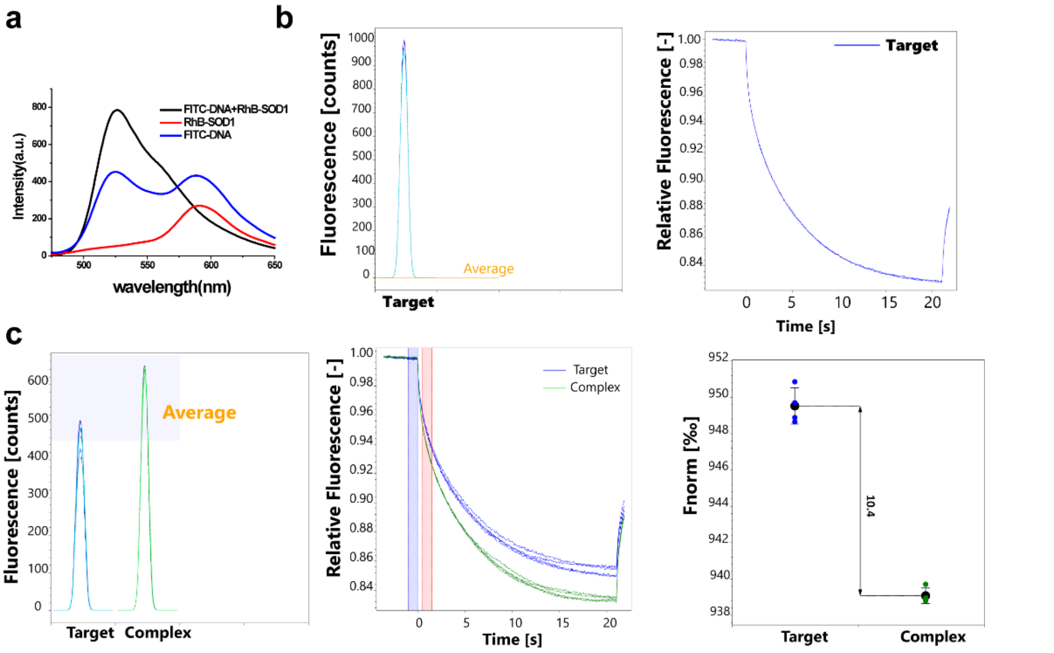

Figure S2. FRET and MST assay of the dsDNA and SOD1. (**a**) 100 µM FITC-dsDNA [1] was mixed with 200 µM RhB-SOD1 at 37 °C for 2 h in 20 mM Tris-HCl (pH 7.4), and the mixture was tested at the excitation wavelength of 450 nm. (**b**) NHS-SOD1 was first diluted to a final concentration of 3 µM by 10 mM pH 7.4 PBS containing 0.05% Tween, and then loaded into the Nano Temper Technologies glass capillaries for MST tests. (**c**) Binding check assays between NHS-SOD1 and dsDNA. 500 nM NHS-SOD1 was mixed with 20 µM dsDNA at room temperature for 5 min, and loaded into the Nano Temper Technologies glass capillaries. MST measurements were carried out using a 20% excitation power. NHS-SOD1 was recognized as the target.

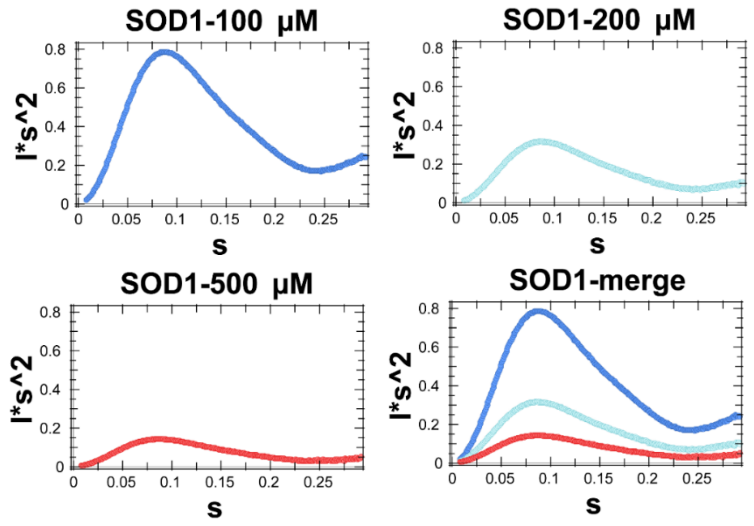

Figure S3. Kratky plot of bovine SOD1.

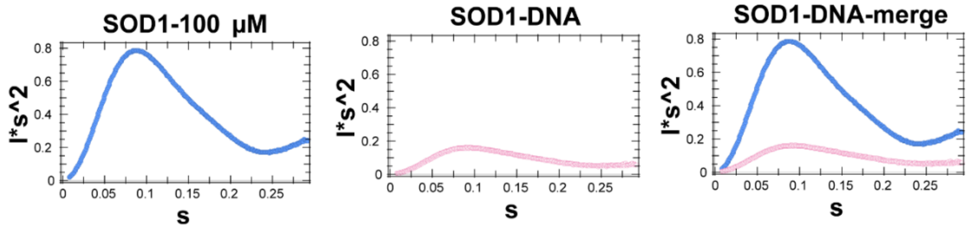

Figure S4. Kratky plot of SOD1 and SOD1-DNA complex.

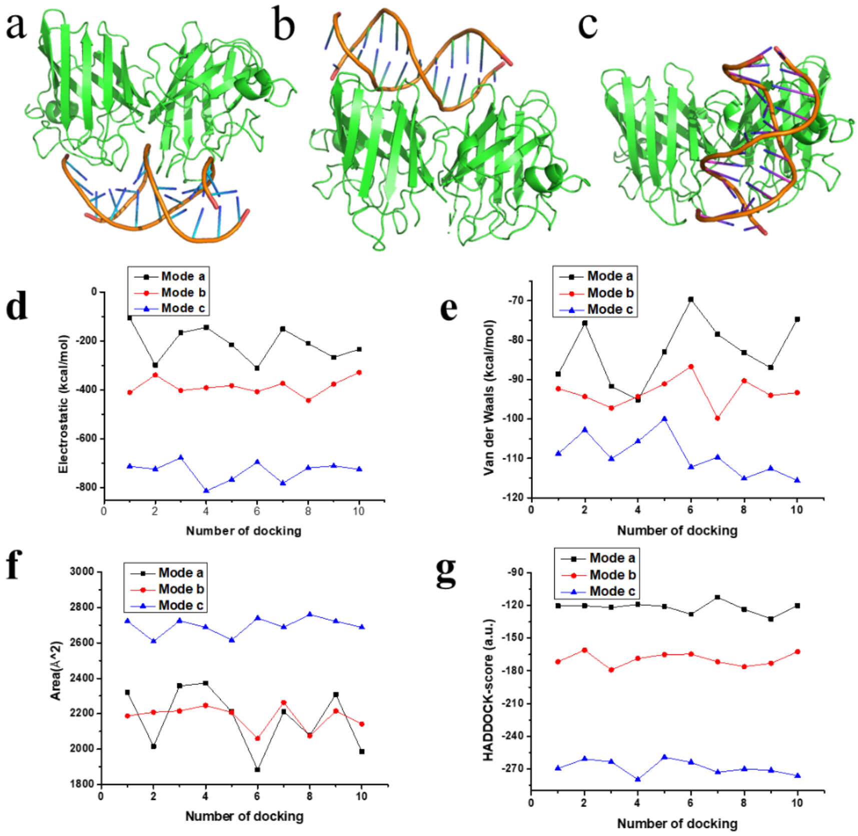

Figure S5. Three binding models (**a**,**b**,**c**) of SOD1 to dsDNA provided by HADDOCK 2.2 and comparisons among three models in the electrostatic and van der Waals energy, contact areas and HADDOCK score (**d**,**e**,**f**,**g**).

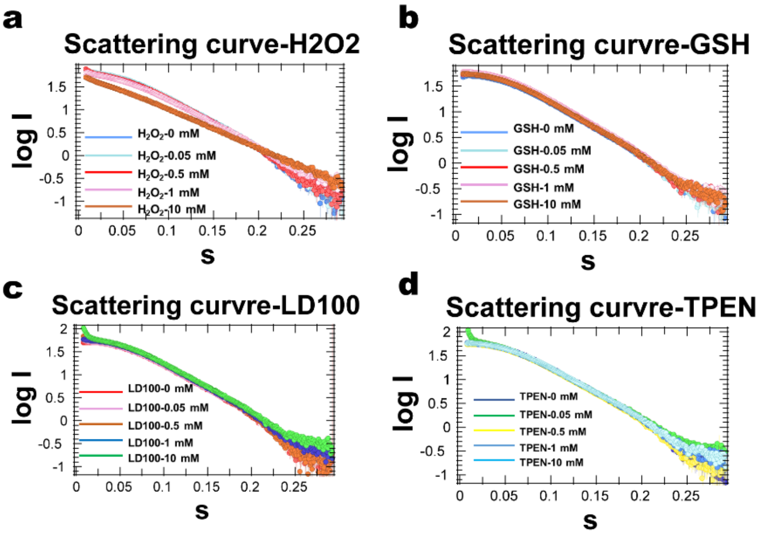

Figure S6. Scattering curves of dsDNA-SOD1 complexes. (**a**) The mixture of SOD1 and dsDNA in the presence of 0, 0.05, 0.5, 1 mM H_2O_2. (**b**) The mixture of SOD1 and dsDNA in the presence of 0, 0.05, 0.5, 1 mM GSH. (**c**) The mixture of SOD1 and dsDNA in the presence of 0, 0.05, 0.5, 1 mM LD100. (**d**) The mixture of SOD1 and dsDNA in the presence of 0, 0.05, 0.5, 1 mM TPEN.

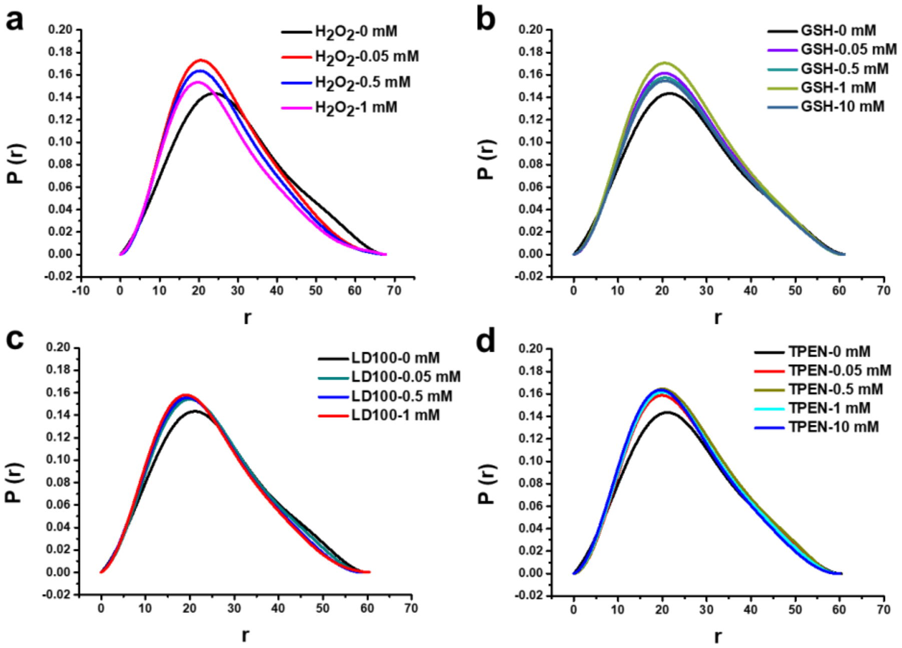

Figure S7. Pair distribution functions of dsDNA-SOD1 complexes computed by GNOM. (**a**) The mixture of SOD1 and dsDNA in the presence of 0, 0.05, 0.5, 1 mM H_2O_2. (**b**) The mixture of SOD1 and dsDNA in the presence of 0, 0.05, 0.5, 1 mM GSH. (**c**) The mixture of SOD1 and dsDNA in the presence of 0, 0.05, 0.5, 1 mM LD100. (**d**) The mixture of SOD1 and dsDNA in the presence of 0, 0.05, 0.5, 1 mM TPEN. Samples were centrifuged at 10000 rpm, 4°C for 10 minutes to remove potential aggregates before data collection.

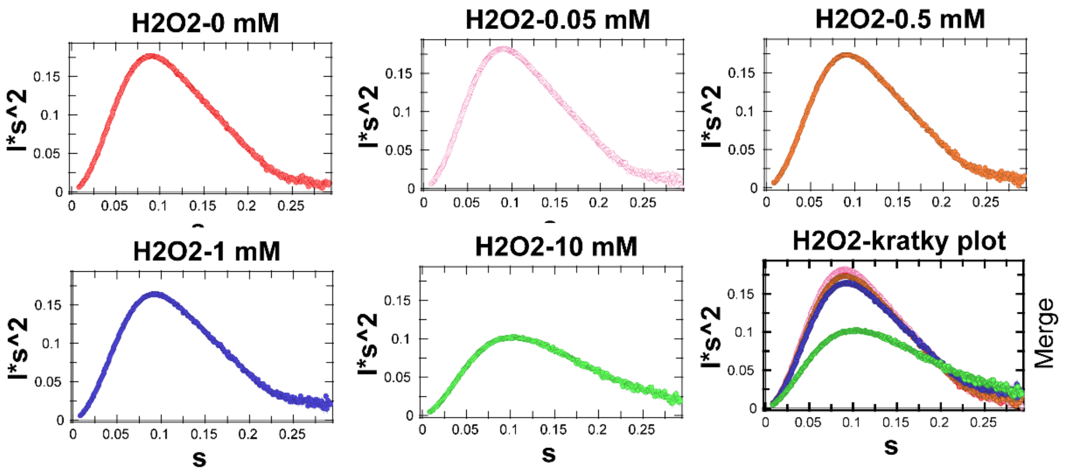

(**a**)

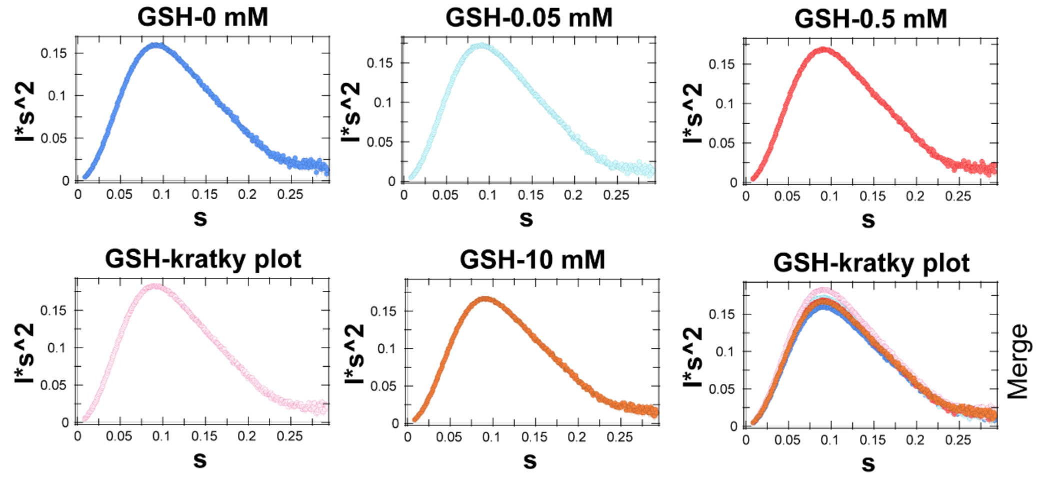

(**b**)

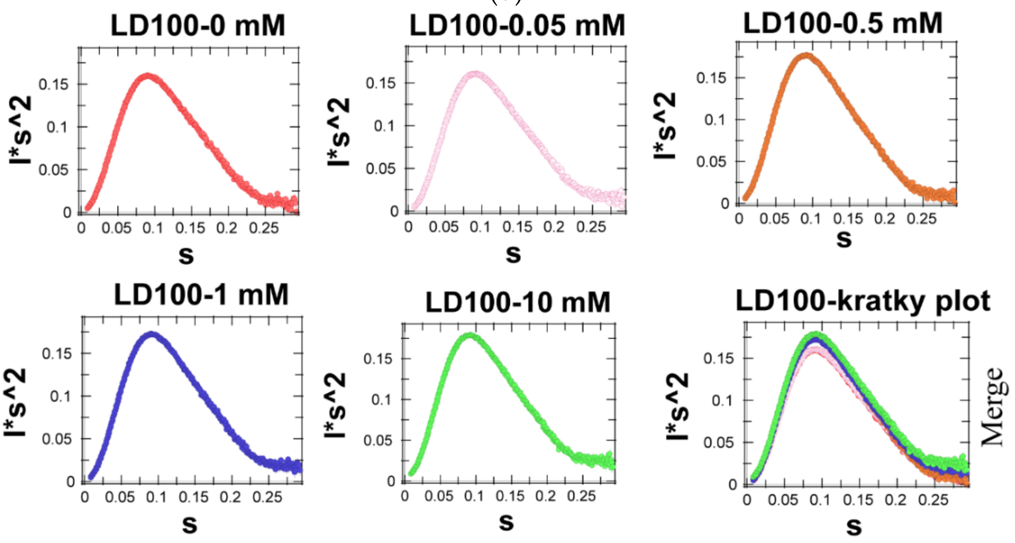

(**c**)

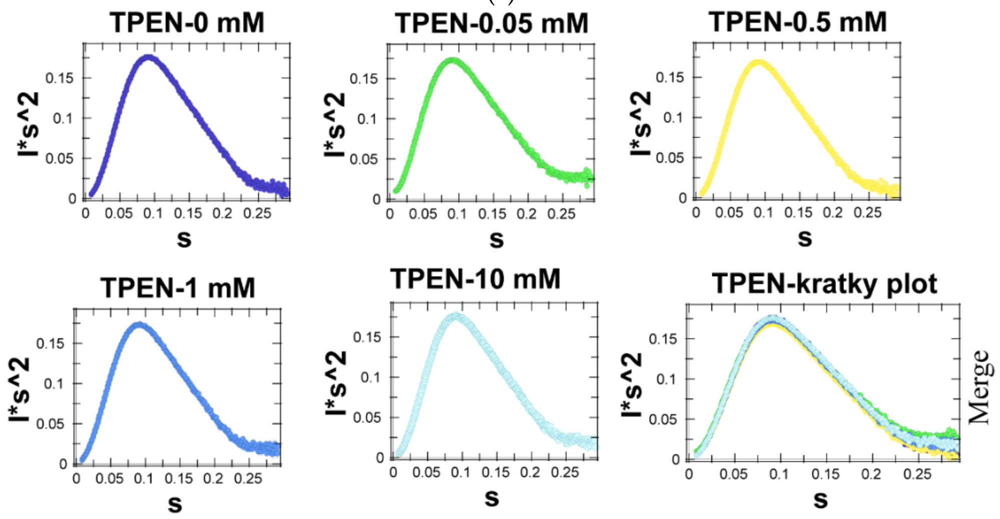

(**d**)

Figure S8. Kratky plot of dsDNA-SOD1 complexes under different conditions (**a**) The mixture of SOD1 and dsDNA in the presence of 0, 0.05, 0.5, 1 mM H_2O_2. (**b**) The mixture of SOD1 and dsDNA in the presence of 0, 0.05, 0.5, 1 mM GSH. (**c**) The mixture of SOD1 and dsDNA in the presence of 0, 0.05, 0.5, 1 mM LD100. (**d**) The mixture of SOD1 and dsDNA in the presence of 0, 0.05, 0.5, 1 mM TPEN.

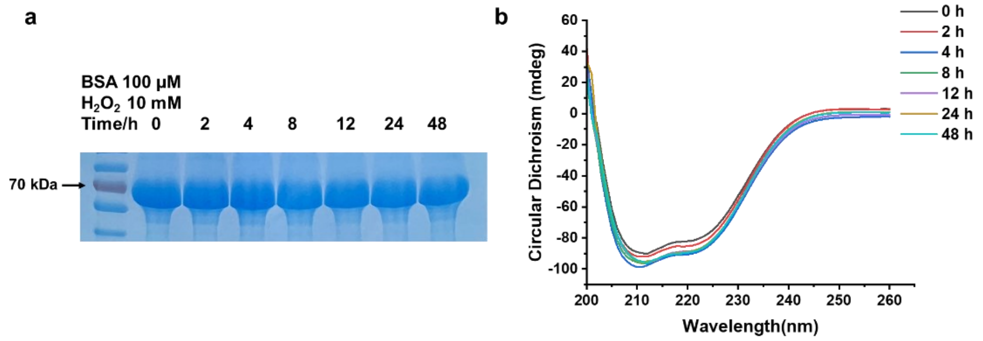

Figure S9. SDS-PAGE and CD spectra of BSA. (**a**) 100 μM BSA was added with 10 mM H_2O_2 for varied time (0, 2, 4, 8, 12, 24 and 48 h) at 37 °C and tested using SDS-PAGE assay. (**b**) 100 μM BSA was added with 10 mM H_2O_2 for varied time (0, 2, 4, 8, 12, 24 and 48 h) at 37 °C and tested by circular dichroism.

References

1. Roy, P.; Rajfur, Z.; Pomorski, P.; Jacobson, K. Microscope-based techniques to study cell adhesion and migration. *Nat. Cell. Biol.* **2002**, *4*, E91–E96.
